# Supplementary material for: Pathogen evasion of social immunity
Source: Nat Ecol Evol. 2023 Feb 2;7(3):450–60. doi: 10.1038/s41559-023-01981-6 (PMC9998270; doi:10.1038/s41559-023-01981-6)
Supplement: Supplementary file 1 — Supplementary Tables 1–3 and Figs. 1–3. [file 41559_2023_1981_MOESM1_ESM.pdf]

nature ecology & evolution

Article https://doi.org/10.1038/s41559-023-01981-6

Pathogen evasion of social immunity

In the format provided by the authors and unedited

Supplementary Tables

Supplementary Table 1) Statistical data analysis of all selection lines. Statistical tests and their results. All p-values are two-sided and corrected following the Benjamini-Hochberg (BH) procedure. Exact p-values and contrasts in the Bayesian analysis are given (except for values <0.0001) and highlighted in bold when p<0.05 or Bayesian level of support LOS >95%. Individual selection treatment abbreviated by I, social selection treatment by S.

**Strain diversity during and after serial passage (Fig. 1b)**				
Wilcoxon Rank Sum Test (all data) :		**W**	**Effect size (η^2)**	**p-value**
**strain number ~ selection treatment**	passage 5	21	0.55	**0.015**
	passage 10	50	0.00	1.000
**Strain composition after serial passage (Fig. 1b)**				
Fisher's Exact Test:				**p-value**
				0.629
**Virulence after serial passage (Fig. 2a)**				
Bayesian multilevel model (all data):		**Dying probability 95 CI**		**HDI lower-upper**
**mortality ~ selection history * current host social context** + (1|strain) + (1|colony), family=bernoulli(), control = list(adapt_delta = 0.99), iter = 5000, chains = 6		SS	0.26	0.14-0.39
		II	0.44	0.28-0.60
		SI	0.3	0.16-0.43
		IS	0.29	0.16-0.43
		**Contrast BH-corrected**		**Level of support (LOS)**
		II vs SS	**0.005**	**99.82**
		II vs IS	**0.005**	**99.92**
		II vs SI	**0.020**	**99.15**
		IS vs SI	0.610	38.73
		IS vs SS	0.410	66.05
		SI vs SS	0.300	79.65
**Spore production after serial passage (Fig. 2b)**				
LMM Overal model (all data):		χ^2	**DF**	**p-value**
**spore number ~ selection history * current host social context** + (1|replicate line) + (1|laboratory stock colony)		21.68	3	**<0.0001**
		**Effect size (η^2)**		
		selection history: 0.64<br>current host social context: <0.0001<br>interaction: 0.04		
Interaction		4.37	1	**0.037**
		**Contrast BH-corrected**		
		II vs SS		**<0.0001**
		II vs IS		0.052
		II vs SI		**0.0007**
		IS vs SI		**<0.0001**
		IS vs SS		**<0.0001**
		SI vs SS		0.340

Supplementary Table 1 continued

Allogrooming elicitation after serial passage (Fig. 3a)				
Wilcoxon Rank Sum Test (all data) :	**W**			**p-value**
**grooming events ~ selection history**	90			**0.0027**
	**Effect size**			
	0.678			
**Allogrooming elicitation by pure ergosterol (Fig. 3c)**				
Wilcoxon Rank Sum Test:	**W**			**p-value**
**grooming events ~ treatment**	155.5			**0.026**
	**Effect size**			
	0.333			
**Allogrooming elicitation by pure cholesterol (Fig. 3d)**				
Wilcoxon Rank Sum Test:	**W**			**p-value**
**grooming events ~ treatment**	345			0.144
	**Effect size**			
	0.214			
**Chemical analysis (Fig. 3b, ED Fig. 1)**				
Permanova:	**F**	**DF**		**p-value**
**compounds ~ selection history, method="eucl"**	4.75	1		**0.021**
Random forest (RF):				
Number of trees: 500<br>No. of variables tried at each split: 6<br>OOB estimate of error rate: 30%				
Wilcoxon RS test on RF-suggested compounds			**W**	**p-value**
	Ergosterol (comp. 18; RI 3269)		82	**0.025**
	compound 7 (RI2631)		85	**0.007**
	compound 17 (RI 3226)		89	**0.007**
	compound 19 (RI 3277)		82	**0.025**
	compound 25 (RI 3420)		79	**0.031**
	compound 16 (RI 3204)		79	**0.031**

Supplementary Table 2) Statistical data analysis of the *M. robertsii* R1-dominant lines. Statistical tests and their results for the subset of the 7 individual and 6 social lines, in which strain R1 was dominant. All p-values are two-sided and corrected following the Benjamini-Hochberg (BH) procedure. Significant p-values ($p<0.05$) and contrasts in the Bayesian analysis (level of support LOS >95%) shown in bold. Individual selection treatment abbreviated by I, social selection treatment by S.

**Virulence after serial passage (Fig. 2a)**			
Bayesian multilevel model (subset R1-dominant lines):	**Dying probability 95 CI**		**HDI lower-upper**
**mortality ~ selection history * current host social context** + (1|strain) + (1|colony), family=bernoulli(), control = list(adapt_delta = 0.99), iter = 4000, chains = 6	SS	0.28	0.10-0.47
	II	0.45	0.24-0.68
	SI	0.28	0.11-0.48
	IS	0.28	0.11-0.47
	**Contrast BH-corrected**		**Level of support (LOS)**
	II vs SS	**0.007**	**99.82**
	II vs IS	**0.002**	**99.92**
	II vs SI	**0.009**	**99.14**
	IS vs SI	0.502	38.73
	IS vs SS	0.491	66.05
	SI vs SS	0.490	79.65
**Spore production after serial passage (Fig. 2b)**			
LMM Overal model (subset R1-dominant lines):	χ^2	**DF**	**p-value**
**spore number ~ selection history * current host social context** + (1|replicate line) + (1|laboratory stock colony)	12.44	3	**0.006**
	**Effect size (η^2)**		
	selection history: 0.55<br>current host social context: 0.07<br>interaction: 0.004		
Interaction	0.95	1	0.329
Selection history	10.65	1	**0.001**
Current host social context	0.33	1	0.562
	**Contrast BH-corrected**		
	II vs SS		**0.0009**
	II vs IS		0.871
	II vs SI		**0.008**
	IS vs SI		**0.008**
	IS vs SS		**0.0009**
	SI vs SS		0.300
**Allogrooming elicitation after serial passage (Fig. 3a)**			
Wilcoxon Rank Sum Test (subset R1-dominant lines):	**W**		**p-value**
**grooming events ~ selection history**	37		**0.026**
	**Effect size**		
	0.638		
**Chemical analysis (Fig. 3b, ED Fig.1)**			
Wilcoxon Rank Sum Test (subset R1-dominant lines):		**W**	**p-value**
	Ergosterol (comp. 18; RI 3269)	34	0.073
	compound 7 (RI2631)	39	**0.020**
	compound 17 (RI 3226)	40	**0.020**
	compound 19 (RI 3277)	34	0.073
	compound 25 (RI 3420)	37	0.053
	compound 16 (RI 3204)	36	0.060

Supplementary Table 3) Chemical spore profiles. Identification number (ID), Kováts retention index (RI) and diagnostic ions, as well as resulting classification (Class), and the confidence level of compound annotations, following the Compound Identification work group of the Metabolomics Society (Online Methods); unknown compounds C1-3 have very similar spectra. Mass spectrum of compound 7 (Unknown A) shown in Supplementary Fig. 3. Note that not every compound was detectable above the signal to noise ratio in each sample.

ID	RI	Diagnostic ions [*m/z*]	Confidence level of annotation	Class
1	1965	129, 213, 256	2	Carboxylic acid
2	2151	55, 264, 282	3	Carboxylic acid
3	2424	67, 262, 280	3	Ester
4	2428	98, 264, 265	3	Ester
5	2473	67, 263, 336	3	Ester
6	2511	134, 239, 299	3	Ester
7	2631	147, 187, 314	4	Unknown A
8	2656	101, 117, 131	4	Unknown B
9	2717	55, 262, 264	3	Ester
10	2820	69, 81, 410	2	Squalene
11	2851	337, 363, 378	3	Ergosterol derivative
12	2903	253, 363, 378	3	Ergosterol derivative
13	3016	105, 251, 376	2	Ergosterol derivative
14	3042	250, 251, 376	3	Ergosterol derivative
15	3053	250, 251, 376	3	Ergosterol derivative
16	3204	253, 363, 396	3	Ergosterol derivative
17	3226	253, 363, 396	3	Ergosterol derivative
18	3269	253, 363, 396	1	Ergosterol
19	3277	251, 376, 394	3	Ergosterol derivative
20	3318	339, 365, 398	3	Ergosterol derivative
21	3333	271, 383, 398	3	Ergosterol derivative
22	3350	189, 218, 424	3	Lupeol derivative
23	3390	175, 218, 426	3	Lupeol derivative
24	3399	393, 411, 426	3	Lupeol derivative
25	3420	99, 283, 417	3	Ester
26	3432	253, 268, 392	3	Ergosterol derivative
27	3440	205, 313, 424	3	Lupeol derivative
28	3463	393, 411, 426	3	Lupeol derivative
29	3467	120, 148, 176	4	Unknown C1
30	3591	205, 313, 424	3	Lupeol derivative
31	3593	120, 148, 176	4	Unknown C2
32	3618	189, 357, 426	3	Lupeol derivative
33	3638	120, 148, 176	4	Unknown C3
34	3659	189, 411, 440	3	Lupeol derivative
35	3681	205, 313, 424	3	Lupeol derivative
36	3687	205, 453, 468	3	Lupeol derivative
37	3730	379, 422, 482	2	Ursane derivative
38	3747	407, 422, 482	3	Lanosterol derivative
39	3761	189, 409, 440	3	Lupeol derivative
40	3783	189, 453, 469	3	Lupeol derivative

Supplementary Figures

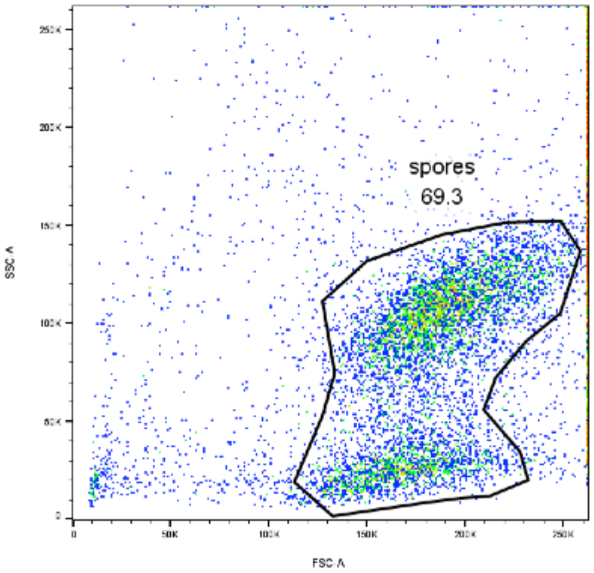

Supplementary Figure 1) Gating strategy in the flow cytometry. To obtain single clones per replicate line (and excluding spore clusters), individual spores were sorted into 96-well plates. Unstained spores were detected using the exemplified gating strategy of FSC/SSC in linear mode (70 µm nozzle). Two spore populations appear, as *Metarhizium* spores are elongated, so that depending on their orientation (horizontal vs. vertical) in the sorter, the forward and sideward scatter values vary slightly. Confirmation that this procedure led to single spores was obtained by microscopy.

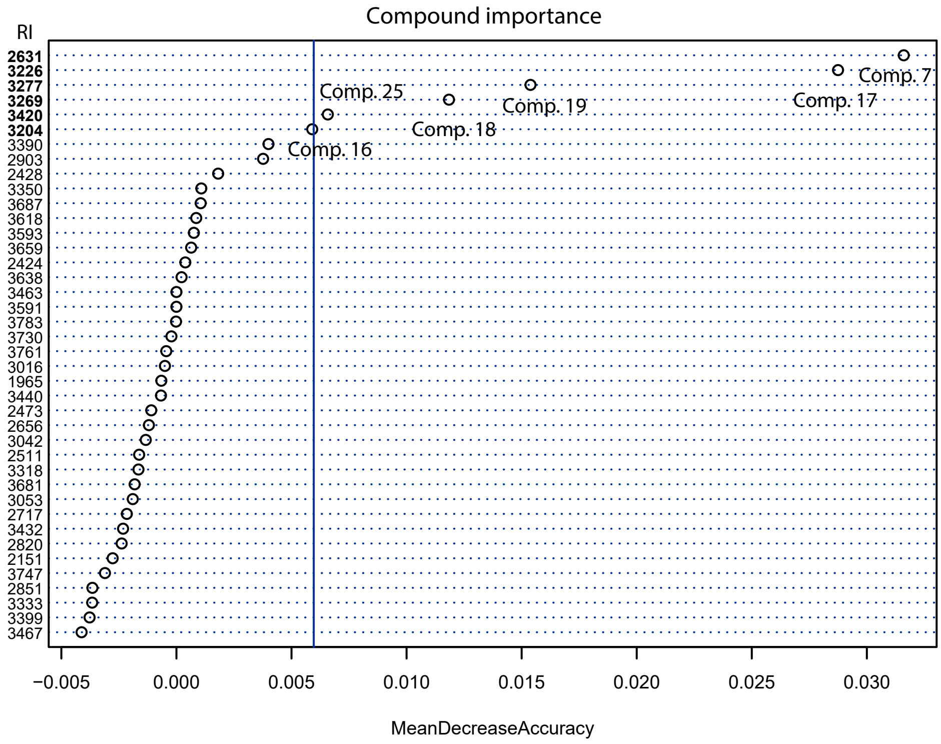

Supplementary Figure 2) Spore compound importance as identified by random forest analysis. Conditional random forest classification (n trees = 500, n variables per split = 6) identified 6 compounds (numbers 7, 16, 17, 18, 19 and 25; Supplementary Table 3) of the chemical spore profiles that contribute to differences between the two selection treatments. Important compounds were selected based on the absolute value of the lowest negative score of the mean decrease in accuracy as a threshold (indicated by the vertical blue line). Compounds are sorted from top to down by their importance of contribution, and named by their Retention Index (RI; Supplementary Table 3).

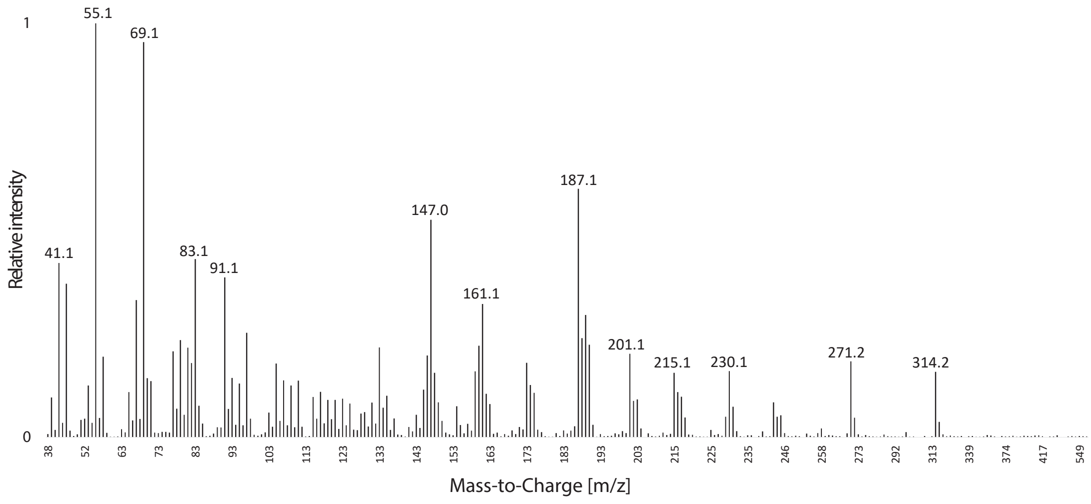

Supplementary Figure 3) Mass spectrum of compound 7. Mass spectrum of compound 7 (Unknown A, Supplementary Table 3), which is significantly lower in abundance in the social than the individual lines (Supplementary Fig. 2, Extended Data Fig. 1).
