# Supplementary material for: Pathogen evasion of social immunity
Source: Nat Ecol Evol. 2023 Feb 2;7(3):450–60. doi: 10.1038/s41559-023-01981-6 (PMC9998270; doi:10.1038/s41559-023-01981-6)
Supplement: Supplementary file 2 — Reporting Summary [file 41559_2023_1981_MOESM2_ESM.pdf]

nature portfolio

Corresponding author(s): Barbara Milutinovic & Sylvia Cremer

Last updated by author(s): Jan 18, 2023

Reporting Summary

Nature Portfolio wishes to improve the reproducibility of the work that we publish. This form provides structure for consistency and transparency in reporting. For further information on Nature Portfolio policies, see our Editorial Policies and the Editorial Policy Checklist.

Statistics

For all statistical analyses, confirm that the following items are present in the figure legend, table legend, main text, or Methods section.

n/a	Confirmed	
☐	☒	The exact sample size (n) for each experimental group/condition, given as a discrete number and unit of measurement
☐	☒	A statement on whether measurements were taken from distinct samples or whether the same sample was measured repeatedly
☐	☒	The statistical test(s) used AND whether they are one- or two-sided<br>*Only common tests should be described solely by name; describe more complex techniques in the Methods section.*
☒	☐	A description of all covariates tested
☐	☒	A description of any assumptions or corrections, such as tests of normality and adjustment for multiple comparisons
☐	☒	A full description of the statistical parameters including central tendency (e.g. means) or other basic estimates (e.g. regression coefficient) AND variation (e.g. standard deviation) or associated estimates of uncertainty (e.g. confidence intervals)
☐	☒	For null hypothesis testing, the test statistic (e.g. F, t, r) with confidence intervals, effect sizes, degrees of freedom and P value noted<br>*Give P values as exact values whenever suitable.*
☐	☒	For Bayesian analysis, information on the choice of priors and Markov chain Monte Carlo settings
☐	☒	For hierarchical and complex designs, identification of the appropriate level for tests and full reporting of outcomes
☐	☒	Estimates of effect sizes (e.g. Cohen's d, Pearson's r), indicating how they were calculated

Our web collection on statistics for biologists contains articles on many of the points above.

Software and code

Policy information about availability of computer code

Data collection	software used for real-time PCR: Bio-Rad CFX Manager software, for droplet digital PCR: QuantaSoft™ Analysis Pro Software (Bio-Rad); for microsatellite analysis: Peak Scanner Software 2; for FACS sorting: Diva 6.2 software; for videos: StreamPix 5 software and VirtualDub software v 1.10.4; for behavioural annotation: BioLogic; for gas chromatography-mass spectrometry: CHRONOS 4.2 software, Axel Semrau and MassHunter Workstation, Data Acquisition software B.07.01 and Qualitative and Quantitative Analysis B.07.00; Agilent Technologies;
Data analysis	statistics performed with R version 4.0.5, packages: DHARMa, bestNormalize, lme4, brms, vegan, randomForest, effectsize, rstatix

For manuscripts utilizing custom algorithms or software that are central to the research but not yet described in published literature, software must be made available to editors and reviewers. We strongly encourage code deposition in a community repository (e.g. GitHub). See the Nature Portfolio guidelines for submitting code & software for further information.

Data

Policy information about availability of data

All manuscripts must include a data availability statement. This statement should provide the following information, where applicable:

- Accession codes, unique identifiers, or web links for publicly available datasets
- A description of any restrictions on data availability
- For clinical datasets or third party data, please ensure that the statement adheres to our policy

All data are provided as source data (Stock_Milutinovic_source_data.xlsx).

Field-specific reporting

Please select the one below that is the best fit for your research. If you are not sure, read the appropriate sections before making your selection.

☐ Life sciences ☐ Behavioural & social sciences ☒ Ecological, evolutionary & environmental sciences

For a reference copy of the document with all sections, see nature.com/documents/nr-reporting-summary-flat.pdf

Ecological, evolutionary & environmental sciences study design

All studies must disclose on these points even when the disclosure is negative.

Study description

We performed a serial passage experiment with fungal pathogens over 10 infection cycles in ant hosts. Exposed ants were either kept alone or attended by two nestmates, to obtain two selection treatments (individual and social host), each performed in 10 replicate lines. We determined the number and identity of fungal strains remaining from the original starting mix of 6 strains for each replicate line at passages 5 and 10 (by line-level quantitative real-time PCR followed by clone-level microsatellite analysis). We compared strain diversity during the course (passage 5) and at the end of the experiment (passage 10) between the selection treatments (by Wilcoxon rank sum tests for independent samples, WRST), as well as the final composition of the strains prevailing at after the serial passage experiment under the two selection treatments (by Fisher exact test). We then characterized the 20 successful lines for their virulence (measured as induced host mortality) and transmission potential (number of spores growing out of the sporulating carcasses) in a common garden experiment (testing the main effects of selection history and current host social context and their interaction, and including replicate line and laboratory stock colony as random effects). We further determined the allogrooming intensity elicited by these fungal lines in nestmate ants (comparing the allogrooming events induced by individual and social lines by Wilcoxon rank sum tests for independent samples, WRST). Characterisation of the chemical spore profiles of the fungal lines by gas chromatography-mass spectrometry revealed lower ergosterol levels in the social lines (Permanova for overall difference between individual and social lines, Random Forest to determine important contributing compounds, WRST for the six identified important compounds, p-value adjustment for multiple testing by the Benjamini Hochberg correction). We therefore performed a bioassay applying ergosterol vs an acetone sham treatment on the ants and determined the elicited allogrooming in nestmates (comparing the allogrooming events in the two treatments by WRST). We further determined the specificity of the allogrooming-elicitation by the pure ergosterol treatment by performing a second bioassay, using the non-fungal, but chemically highly similar compound cholesterol vs sham application.

Research sample

As pathogen, we used 6 strains of the entomopathogenic fungus Metarhizium, all collected from the same sampling site and hence representing a natural, sympatric fungal community (3 strains of M. robertsii and 3 strains of M. brunneum), making natural coinfections of these strains likely. As host, we used workers of the Argentine ant, Linepithema humile, which are susceptible to all the six fungal strains. Queens, workers and brood were collected in 2011 and 2016 from the wide-ranging "main" supercolony that L. humile forms in large areas of Southern Europe (as a characteristic of invasive ants, nests are interconnected to supercolonies). Our sampling site was close to Sant Feliu de Guíxols, Spain (N 41° 49', E 3° 03'). The field-collected insects were brought back to the laboratory to set up large stock colonies, out of which workers were taken from outside the brood chambers for use in the experiments.

Sampling strategy

We ran our serial passage experiment in 10 replicate lines each of the individual and social host selection treatment, to obtain a high enough replication to be able to test for significant differences between the two treatments (as most statistical tests require a minimum of 6 replicates per treatment group). For each line at each passage, we pooled the outgrowing spores from the first 8 carcasses, which was sufficient to obtain enough spores to expose the ants for the next infection cycle without the need for additional expansion on the plate (which, due to strain-strain competition could have altered pathogen strain composition), and to freeze remaining spores for molecular analysis and clone individualisation; the latter allowed for growth of the successful lines for characterization of their virulence and transmission (in the common garden experiment), as well as their allogrooming elicitation and chemical profiles. To obtain these 8 carcasses throughout the serial passage experiment for each replicate line and passage in both of the treatments, a total of 6312 fungus-exposed ants and 8026 nestmates was used, based on preliminary assessments of mortality induction and carcass spore outgrowth. In the common garden experiment, 19-20 ants per replicate line from each of the four combinations of selection history and the current social host condition (total 797 ants) were exposed, to allow for a reliable estimation of the proportion of ants dying per replicate line and enough outgrowing carcasses for spore number quantification (median of 5 carcasses per combination, total 215). To ensure a large enough sample size for the induction of allogrooming in the 20 lines, we quantified nestmate allogrooming in three biological replicates per line. To assure reliable read-out of the gas chromatography-mass spectrometry, we ran each line in three technical replicates. As we tested for the effect of only a single compound from the natural spore profile in the bioassay, the relatively high sample size of >20 ants each receiving the ergosterol and sham treatment was chosen. The same was true for the bioassay with the non-fungal-derived cholesterol.

Data collection

The serial passage and common garden experiments including the molecular strain characterisations were performed as described in the study description and sampling strategy by MS and AVG. Chemical data, including running of the spore extracts in the gas chromatograph – mass spectrometer, quantitative analysis of the abundance per peak and the compound identification from the mass spectrum were obtained by FW, NK, MH and TS. The fungal line elicitation of allogrooming was observed by MN. MH performed the bioassays.

Timing and spatial scale

The serial passage experiment was run over a duration of ½ year in 2011-2012, followed by the molecular analysis of the strains and clones, so that the common garden experiment and the allogrooming elicitation experiment could be performed in 2013. All these experiments used the ants collected from the field in Spain in 2011 and subsequently reared in the laboratory. In 2015, the fungal lines were run on a gas chromatograph-mass spectrometer, followed by analysis of the spore profiles for their qualitative (mass spectra) and quantitative (peak integration) compound characteristics and statistical differences between the strains, revealing lower ergosterol amounts in the social than individual lines. The bioassay testing for the grooming-eliciting effect of application of pure

ergosterol was performed in 2021 using ants sampled in 2016 during a second ant collection from the same field population in Spain. The specificity bioassay with pure cholesterol was performed in 2022 with ants collected the same year, again from the same population.

Data exclusions	We excluded a single value from our analysis, as we identified an outlier during the carcass spore counts (from replicate I5), which had a value that was 100-fold higher than the spore counts of the other carcasses of its replicate, suggesting a counting error in this single carcass. We tested whether exclusion of this outlier value had an effect on our analysis by rerunning the statistics including this value, which did not change the statistical outcome.
Reproducibility	Experiments were run in simultaneous replication, i.e. in the serial passage experiment, 10 independent replicate lines were obtained in parallel for each of the selection treatments. As our molecular analysis revealed that 7/10 individual and 6/10 social lines had the same strain as winner strain, we also performed a statistical analysis of only this subset, which showed the robustness of the effects of the individual vs social host selection treatment independent of fungal strain identity. The allogrooming elicitation was replicated three times per fungal line (3 biological replicates). In the GC-MS, each fungal line was run in 3 technical replicates, revealing high reproducibility of the method. For the bioassays, we chose a sample size of n>20 per treatment.
Randomization	For all experiments, workers were picked randomly from outside the brood chambers in the laboratory stock colonies, in which the ants were reared after field collection (with laboratory stock colony included as random effect in the linear models). These workers were then randomly assigned to treatment (e.g. exposed ant vs nestmate, ergosterol- / resp. cholesterol-treatment vs sham-treatment). During video acquisition of the allogrooming elicitation, the three replicates per fungal line were randomised over the cameras. For the gas-chromatography-mass spectrometry, spore extracts were run in a randomised manner, intermingled with blank runs and handling controls. In the bioassays, the treatments were randomised during video acquisition.
Blinding	The observers annotating the videos for allogrooming were blinded for treatment to assure bias-free observation. In detail, in the elicitation of allogrooming from fungal lines, the observer was blind for both the selection history, as well as the individual ant treatment, and in the bioassay blind for the treatment (ergosterol, respectively cholesterol, vs sham) of the treated individual.

Did the study involve field work? ☐ Yes ☒ No

Reporting for specific materials, systems and methods

We require information from authors about some types of materials, experimental systems and methods used in many studies. Here, indicate whether each material, system or method listed is relevant to your study. If you are not sure if a list item applies to your research, read the appropriate section before selecting a response.

Materials & experimental systems

n/a	Involved in the study
☒	☐ Antibodies
☒	☐ Eukaryotic cell lines
☒	☐ Palaeontology and archaeology
☐	☒ Animals and other organisms
☒	☐ Human research participants
☒	☐ Clinical data
☒	☐ Dual use research of concern

Methods

n/a	Involved in the study
☒	☐ ChIP-seq
☐	☒ Flow cytometry
☒	☐ MRI-based neuroimaging

Animals and other organisms

Policy information about studies involving animals; ARRIVE guidelines recommended for reporting animal research

Laboratory animals	The insects (ants) used in our experiments were collected from the field and reared in the laboratory before use in the experiments.
Wild animals	We used workers of the invasive Argentine ant, Linepithema humile, in our experiments. Queens, workers and brood from the main European supercolony were collected from their nests in the soil from a field population close to Sant Feliu de Guíxols, Spain (N 41° 49’, E 3° 03’) in 2011, 2016 and 2022. Ants were transported back to the laboratory in plastic boxes, to be reared as large stock colonies in plastic boxes containing smaller nest boxes and a plastered floor. Ants either died from fungal infection during the experiments or were frozen at the end of the experiment.
Field-collected samples	Stock colonies were reared in an incubator at 27 °C with day/night light cycle, which assured high productivity of the colonies as the experiments required > 15,000 workers. The experiments were carried out under conditions optimal for Metarhizium to establish infections in the ant, in a temperature- and humidity-controlled room at 23 °C, 65% RH and a 12h day/night light cycle. During experiments, ants were kept in petri dishes with plastered floor and provided with ad libitum access to a sucrose-water solution (100g/L) and plaster was watered every 2-3 days to keep humidity high.
Ethics oversight	We used ants (insects, invertebrates) as our study animals. The study was performed on workers of an invasive ant species, the Argentine ant, Linepithema humile. Collection of this unprotected species from the field was in compliance with international regulations, such as the Convention on Biological Diversity and the Nagoya Protocol on Access and Benefit-Sharing (ABS; permits not required for collections before 2017; 2022: ABSCH-IRCC-ES-260624-1 ESNC126 and SF0171/22). Transport to and rearing of the ants in the laboratory, as well as all experimental work followed European and Austrian law and institutional ethical guidelines of ISTA (Institute of Science and Technology Austria).

Note that full information on the approval of the study protocol must also be provided in the manuscript.

Flow Cytometry

Plots

Confirm that:

- [ ] The axis labels state the marker and fluorochrome used (e.g. CD4-FITC).
- [ ] The axis scales are clearly visible. Include numbers along axes only for bottom left plot of group (a 'group' is an analysis of identical markers).
- [ ] All plots are contour plots with outliers or pseudocolor plots.
- [ ] A numerical value for number of cells or percentage (with statistics) is provided.

Methodology

Sample preparation	We used flow cytometry to individualise the spores from the spore pools collected from the sporulating carcasses as described in the sampling strategy. Hence, pure spore suspensions of the harvested spores per replicate line, but no additional cell populations, constituted the starting population for flow cytometry, which were then separated into individual clones.
Instrument	FACS ARIA III, BD Biosciences
Software	Diva 6.2 software
Cell population abundance	Each sorted population only contained the spores of the respective fungal line. The aim of flow cytometry was not to choose a particular subpopulation, but to sort our lines as single spores into 96-well plates containing agar in each well (as an effective and reliable alternative to streaking out the spores on agar), where they grew into colony forming units (CFUs). These individual spore clones were used for (i) microsatellite analysis to obtain strain identity, and (ii) for growth expansion of the fungal lines to allow characterisation of their virulence, transmission, allogrooming elicitation and chemical profiles (growing from individual spores was required as growth of a spore pool on agar plates leads to growth inhibition of some strains due to strain-strain competition).
Gating strategy	The unstained spore populations were detected using the FSC (Forward Scatter) / SSC (Side Scatter) in linear mode (70 µm nozzle). Purity mode was set to ‘single cell’ and spore clones were obtained by sorting one particle event into each well. This procedure allowed to grow mono-clone cultivars, as spores were sorted into separate wells, and cases of spores sticking together as e.g. duplets or triplets were excluded (see Supplemental Fig. 1 as an example of the gating strategy). During method establishment, it was verified that the chosen procedure led to single spores by performing both visual checking under the microscope and by reanalysis of the sorted population.

- [x] Tick this box to confirm that a figure exemplifying the gating strategy is provided in the Supplementary Information.
